# The Effects of Leaf Roughness, Surface Free Energy and Work of Adhesion on Leaf Water Drop Adhesion

**DOI:** 10.1371/journal.pone.0107062

**Published:** 2014-09-08

**Authors:** Huixia Wang, Hui Shi, Yangyang Li, Yanhui Wang

**Affiliations:** 1 Institute of Forest Ecology, Environment and Protection, Chinese Academy of Forestry, Beijing, China; 2 School of Environmental and Municipal Engineering, Xi'an University of Architecture and Technology, Xi'an, China; 3 Institute of Soil and Water Conservation, Northwest A & F University, Yangling, Shaanxi, China; Harbin Institute of Technology, China

## Abstract

The adhesion of water droplets to leaves is important in controlling rainfall interception, and affects a variety of hydrological processes. Leaf water drop adhesion (hereinafter, adhesion) depends not only on droplet formulation and parameters but also on the physical (leaf roughness) and physico-chemical (surface free energy, its components, and work-of-adhesion) properties of the leaf surface. We selected 60 plant species from Shaanxi Province, NW China, as experimental materials with the goal of gaining insight into leaf physical and physico-chemical properties in relation to the adhesion of water droplets on leaves. Adhesion covered a wide range of area, from 4.09 to 88.87 g/m^2^ on adaxial surfaces and 0.72 to 93.35 g/m^2^ on abaxial surfaces. Distinct patterns of adhesion were observed among species, between adaxial and abaxial surfaces, and between leaves with wax films and wax crystals. Adhesion decreased as leaf roughness increased (*r* =  −0.615, *p* = 0.000), but there were some outliers, such as *Salix psammophila* and *Populus simonii* with low roughness and low adhesion, and the abaxial surface of *Hyoscyamus pusillus* and the adaxial surface of *Vitex negundo* with high roughness and high adhesion. Meanwhile, adhesion was positively correlated with surface free energy (*r* = 0.535, *p* = 0.000), its dispersive component (*r* = 0.526, *p* = 0.000), and work of adhesion for water (*r* = 0.698, *p* = 0.000). However, a significant power correlation was observed between adhesion and the polar component of surface free energy (*p* = 0.000). These results indicated that leaf roughness, surface free energy, its components, and work-of-adhesion for water played important roles in hydrological characteristics, especially work-of-adhesion for water.

## Introduction

The retention of water drops by leaves can be measured as the amount of surface water per unit leaf area at a point which additional water can no longer be retained and starts to drip off [1]. Leaf water drop adhesion (hereinafter, adhesion) varies among species from 0.1 to 500 g/m^2^ [1]–[3]. Plants may benefit from low adhesion via, for example, decreased interception of water by the canopy facilitating water reaching the soil as well as improved plant water balance in arid or semi-arid environments [4], [5], decreased dry and wet deposition [6] and decreased pathogen infection rates [7], [8]. However, surfactants are often added to water during pest and foliar fertilizer management, to modify the surface tension of the spray droplet, allowing it to adhere and spread on leaf surfaces. The different degree of adhesion is determined by the work-of-adhesion for water [9]. The spread of liquids on a particular leaf surface is dependent on the leaf wettability or hydrophobicity of the leaf itself [10]. These properties vary among species and are, to some extent, influenced by factors such as leaf age [11], [12], leaf surface (adaxial/abaxial) [12], epiphytic microorganisms [13], and environmental conditions [14].

The wettability or hydrophobicity of a surface is characterized by the static contact angle between a water droplet and the surface [4], [15]. Nanko et al. [16] and Nairn et al. [10] estimated the ratio of mean water droplet adhesion values over a range of drop sizes and leaf surfaces. Holder [5] and Haines et al. [17] observed the amount of water retained on leaf surfaces; both found that one factor governing water retention was leaf wettability or hydrophobicity. The factors that govern leaf wettability or hydrophobicity need to be quantified if leaf water retention in modeled. These factors can be divided into two categories: physical and chemical interactions between the leaf surface and water droplets [10], [18]. Thus far, few studies have focused on isolating and quantifying each of these properties; instead, most studies quantitatively measuring behaviors that are allied to wettability or hydrophobicity (such as contact angles).

In previous studies, researchers found that plant species with rough leaves caused by epicuticular wax crystals and epidermal cells retained fewer droplets [19], [20]. A layer of epicuticular wax always covers leaf surfaces, whether in the form of amorphous films or wax crystals with micro- and nano-structures. A mixture of compounds make up of these waxes; the amount, form, composition, and constituents of waxes are highly characteristic for a given plant species, plant part, or developmental stage. Typically, these waxes have a hydrocarbon backbone with 20 to >40 carbon atoms, including *n*-alkanes and smaller portions of iso- and anteiso-homologues, primary alcohols, fatty acids, aldehydes, secondary alcohols, ketones, *ß*-diketones, and *n*-alkyl esters [18], [21]. The wettability or hydrophobicity of the chemical components results from the effect of the chemical nature of the epicuticular waxes that could facilitate interactions between the chemical functional groups on the leaf surface and the droplet molecules owing to the presence of functional groups [10], [18], [22]. Different plant species have different wax compositions and constituents and, as a result, exhibit differences in leaf surface free energy. According to Fowkes [23] and Owens-Wendt [24], leaf surface free energy can be divided into two components: dispersive and polar. These authors considered only these two components of the leaf surface free energy.

This study used 60 plant species in Shaanxi Province, NW China, and quantified leaf contact angles and adhesion on the adaxial and abaxial surfaces of leaves. Observations of scanning electron microscope images and use of the thermodynamic approach [23]–[26] were used to analyze the physical (roughness) and physico-chemical (surface free energy, its components, and work-of-adhesion) properties of leaves of 60 species. The goal was to determine and quantify the contribution of leaf physical and physico-chemical aspects to the properties of adhesion. This study will help researchers to understand the roles of leaf physical and physico-chemical surface properties that influence water balances in forest areas and to characterize moisture distribution.

## Materials and Methods

### Ethics statement

The sampling sites, Chunhua, Yichuan, and Shenmu, are parts of field experimental stations of the Institute of Soil and Water Conversation, Northwest A & F University or the ecological field practice stations of Xi'an University of Architecture and Technology, and are available for teaching and research of their respective institutions and universities. Leaf sampling did not involve any endangered or protected species, and all the species we investigated were common in NW China.

### Plant materials and leaf sampling

A total of 60 plant species were selected for this study (Table 1); each was selected because it was common in the study area.

**Table 1:** Sixty species used to measure leaf water drop adhesion, surface roughness, contact angle of water (*θ_w_*) and diiodomethane (*θ_d_*).

Species	Families	Leaf habit	Life form	Unit leaf area (cm^2^)	Wax form		Trichomes		Leaf roughness		*θ_w_*		*θ_d_*		Leaf water drop adhesion (g/m^2^)	
					AD	AB	AD	AB	AD	AB	AD	AB	AD	AB	AD	AB
*Agrimonia pilosa*	Rosaceae	Deciduous	Herb	28.81±3.68	Film	Film	−	−	2	2	69.0±9.2	58.2±8.2	55.3±5.9	49.7±8.6	66.24±12.96	93.35±10.32
*Agropyron mongolicum*	Gramineae	Deciduous	Herb	7.79±1.34	Crystal	Crystal	−	−	5	5	138.0±5.0	99.2±8.9	103.5±6.4	85.9±4.8	9.16±2.33	3.93±1.32
*Amorpha fruticosa*	Leguminosae	Deciduous	Shrub	2.45±0.46	Crystal	Crystal	+	+	5	5	134.9±7.6	135.0±4.8	78.2±12.2	98.6±4.9	11.24±0.92	8.97±1.24
*Amygdalus davidiana*	Rosaceae	Deciduous	Shrub	7.99±2.58	Film	Crystal	−	−	3	4	79.0±26.2	117.2±12.6	61.7±4.3	80.8±7.7	47.67±3.40	28.42±3.24
*Anemone vitifolia*	Ranunculaceae	Deciduous	Herb	160.26±24.16	Film	−	+	+	2	5	102.5±9.5	141.0±4.1	62.8±6.0	112.0±8.0	46.01±2.37	11.77±0.68
*Armeniaca sibirica*	Rosaceae	Deciduous	Tree	11.16±3.07	Film	Film	−	−	3	3	66.2±13.6	100.8±4.6	50.0±5.2	81.6±9.8	32.44±6.34	13.67±2.59
*Artemisia desertorum*	Compositae	Deciduous	Shrub	0.30±0.05	Film	Film	−	−	3	3	63.8±12.4	61.5±12.2	47.4±10.1	55.3±7.0	30.08±10.61	
*Artemisia dubia*	Compositae	Deciduous	Herb	8.10±2.81	−	−	+	+	5	5	134.0±12.5	140.4±3.0	73.3±8.1	116.7±4.1	10.39±5.40	11.27±2.14
*Artemisia gmelinii*	Compositae	Deciduous	Herb	5.96±1.17	Film	−	+	+	3	5	104.9±10.5	140.8±6.8	68.8±7.1	108.2±6.2	40.34±3.96	14.56±0.85
*Astragalus adsurgens*	Leguminosae	Deciduous	Herb	1.66±0.23	Crystal	Crystal	+	+	5	3	137.5±4.1	128.4±6.5	101.8±6.5	100.7±7.7	4.09±0.42	5.37±1.78
*Betula dahurica*	Betulaceae	Deciduous	Tree	16.44±2.29	Crystal	Crystal	−	−	3	5	82.7±12.6	81.5±21.2	62.5±3.6	75.7±8.1	44.28±4.67	42.34±2.87
*Betula platyphylla*	Betulaceae	Deciduous	Tree	16.44±2.29	Crystal	Crystal	−	−	3	5	91.0±6.8	63.3±17.6	55.7±7.3	51.3±3.7	25.48±2.03	31.09±5.75
*Buddleja alternifolia*	Betulaceae	Deciduous	Shrub	6.47±1.02	Film	−	+	+	3	5	87.7±15.9	137.0±6.8	60.4±6.7	107.0±3.2	32.01±7.15	18.13±3.24
*Bupleurum*	Umbelliferae	Deciduous	Herb	6.22±2.00	Crystal	Crystal	−	−	5	5	127.7±3.7	128.0±7.1	98.3±8.9	94.8±4.0	12.59±2.58	10.00±0.52
*Caragana korshinskii*	Leguminosae	Deciduous	Shrub	0.75±0.02	Film	Film	+	+	5	5	133.7±7.3	132.0±6.7	85.0±11.2	79.4±13.2	14.24±1.29	13.70±2.83
*Carduus nutans*	Compositae	Deciduous	Herb	28.16±6.79	Film	Crystal	+	+	2	5	94.7±9.2	139.5±5.3	55.9±4.6	111.1±4.3	88.87±4.06	0.72±0.19
*Carex callitrichos*	Cyperaceae	Deciduous	Herb	3.32±0.53	Film	Crystal	−	−	4	5	84.0±10.7	77.3±8.9	53.5±3.2	56.2±5.5	56.63±14.22	18.30±3.68
*Chloris virgata*	Gramineae	Deciduous	Herb	7.79±1.34	Crystal	Crystal	+	+	5	5	136.9±5.1	125.3±5.3	98.9±6.4	82.7±7.5	6.15±2.42	3.98±2.42
*Cleistogenes chinensis*	Gramineae	Deciduous	Herb	1.09±0.25	Crystal	Crystal	+	+	5	5	116.2±7.2	137.0±5.2	90.5±12.2	74.6±7.4	5.57±0.37	18.72±2.42
*Cotoneaster acutifolius*	Rosaceae	Deciduous	Shrub	3.23±0.64	Crystal	Crystal	+	+	4	5	125.7±10.5	116.1±15.8	102.4±4.4	107.4±9.8	21.98±2.80	40.28±8.02
*Cynanchum chinense*	Asclepiadaceae	Deciduous	Herb	16.10±2.72	Crystal	Crystal	+	+	5	5	132.9±6.7	137.7±8.3	87.6±9.9	101.4±6.4	19.57±0.90	17.12±1.44
*Cynanchum komarovii*	Asclepiadaceae	Deciduous	Herb	5.06±1.54	Film	Film	−	−	2	2	62.1±11.9	63.2±16.6	51.3±2.8	50.1±3.8	38.38±3.75	5.39±0.89
*Elaeagnus pungens*	Elaeagnaceae	Evergreen	Shrub	7.88±1.74	Film	Film	−	+	3	4	80.9±22.5	99.7±17.9	61.1±8.0	64.2±4.8	54.77±6.73	24.99±3.54
*Erodium stephanianum*	Geraniaceae	Deciduous	Herb	14.04±3.52	Film	Film	+	+	2	2	98.4±12.7	111.9±9.9	76.2±8.3	84.6±13.0	70.78±4.60	10.42±1.67
*Euphorbia lunulata*	Euphorbiaceae	Deciduous	Herb	2.12±0.43	Crystal	Crystal	−	−	5	5	130.7±7.6	121.2±5.2	75.1±9.8	73.5±9.1	16.87±3.61	12.87±2.27
*Forsythia suspense*	Oleaceae	Deciduous	Shrub	3.64±0.93	Film	Film	−	−	4	4	74.5±10.8	73.0±6.1	67.5±4.1	52.1±2.4	45.33±2.52	34.73±9.40
*Hedysarun mongolicum*	Papilionaceae	Deciduous	Shrub	1.31±0.26	Crystal	Crystal	+	+	5	3	128.9±4.9	88.5±15.7	96.2±7.7	63.3±4.2	22.18±4.87	13.64±3.74
*Hippophae rhamnoides*	Elaeagnaceae	Deciduous	Shrub	1.92±0.35	Film	−	−	−	3	4	99.5±4.0	102.2±5.9	60.7±3.8	63.0±5.1	67.53±4.65	51.66±8.32
*Hyoscyamus pusillus*	Solanaceae	Deciduous	Herb	22.43±4.58	Film	Film	+	+	4	4	53.8±23.9	56.7±22.0	40.8±3.5	41.3±2.3	43.76±5.57	87.24±10.01
*Ixeris polycephala*	Compositae	Deciduous	Herb	14.62±6.55	Crystal	Crystal	−	−	5	5	133.9±6.2	140.4±7.3	95.1±7.5	96.2±5.4	9.80±0.90	3.76±0.87
*Koelreuteria paniculata*	Sapindaceae	Deciduous	Tree	16.14±6.04	Film	Film	+	+	2	3	88.1±13.6	82.8±8.9	65.6±4.2	63.3±5.3	42.96±8.46	31.64±7.47
*Lespedeza bicolor*	Leguminosae	Deciduous	Herb	1.55±0.42	Crystal	Crystal	−	+	5	5	126.1±5.7	129.7±4.6	87.6±9.6	101.6±3.9	13.14±7.68	18.48±3.69
*Leymus chinensis*	Gramineae	Deciduous	Herb	5.18±1.19	Crystal	Crystal	−	−	5	5	130.4±6.7	101.7±7.9	102.8±5.6	80.3±7.6	8.49±6.57	30.36±6.30
*Lonicera hispida*	Caprifoliaceae	Deciduous	Shrub	1.97±0.45	Film	Film	+	+	3	2	67.0±20.9	98.7±9.1	47.1±4.5	54.6±3.0	70.93±6.80	72.93±9.93
*Medicago sativa*	Leguminosae	Deciduous	Herb	0.80±0.07	Crystal	Crystal	+	+	5	5	128.8±5.9	132.6±8.3	101.4±9.5	102.9±6.1	8.62±3.17	6.08±2.25
*Melilotus officinalis*	Leguminosae	Deciduous	Herb	3.45±0.91	Crystal	Crystal	+	+	5	5	125.8±5.8	123.4±5.7	92.6±8.1	87.0±8.0	29.08±2.24	11.94±0.90
*Ostryopsis davidiana*	Betulaceae	Deciduous	Shrub	11.16±2.29	Film	Crystal	+	+	3	5	91.0±23.1	44.6±10.6	50.8±5.3	42.0±4.9	39.31±5.44	80.17±13.79
*Oxytropis psammocharis*	Leguminosae	Deciduous	Herb	0.06±0.01	Film	Crystal	+	+	4	4	138.6±9.9	133.3±6.0	95.9±10.0	41.7±15.0	7.29±1.28	4.46±1.29
*Parinia heterophylla*	Valerianaceae	Deciduous	Herb	7.82±3.27	Film	Film	−	−	2	2	60.9±9.2	95.2±12.3	57.8±6.6	61.1±9.3	54.69±4.97	45.78±21.48
*Pinus tabulaeformis*	Pinaceae	Evergreen	Tree	6.98±0.53	Film	Film	−	−	3	3	66.5±4.2	62.6±8.5	42.6±5.3	39.8±3.9	75.76±12.53	
*Poa sphondylodes*	Gramineae	Deciduous	Herb	10.22±1.13	Crystal	Crystal	+	+	5	5	132.1±7.0	112.5±6.7	99.3±6.5	64.9±8.1	4.31±0.98	2.07±0.88
*Populus davidiana*	Salicaceae	Deciduous	Tree	14.01±4.25	Crystal	Crystal	−	−	5	5	119.3±13.2	131.2±8.0	79.0±7.5	109.1±2.7	26.28±4.41	10.17±±5.39
*Populus simonii*	Salicaceae	Deciduous	Tree	12.30±2.20	Film	Film	−	−	1	1	80.0±11.9	86.2±9.0	51.4±3.0	57.2±2.5	31.34±2.20	38.62±6.41
*Potentillae chinensis*	Rosaceae	Deciduous	Herb	17.48±2.53	Film	−	−	+	2	5	98.5±13.7	135.1±8.3	50.2±11.7	97.9±10.6	53.16±4.58	10.73±1.55
*Potentilla supine*	Rosaceae	Deciduous	Herb	6.23±1.41	Film	Film	−	+	2	3	107.2±16.0	94.1±15.8	60.2±5.5	58.3±4.9	59.86±9.88	81.38±4.71
*Pyrus betulifolia*	Rosaceae	Deciduous	Tree	9.71±2.44	Film	Film	−	−	3	4	95.2±5.4	97.2±4.1	60.8±3.3	59.2±4.3	22.86±3.67	17.74±3.27
*Quercus wutaishanica*	Fagaceae	Deciduous	Tree	21.20±6.00	Crystal	Crystal	−	−	4	4	88.9±24.5	132.0±4.6	63.0±6.3	94.6±5.6	48.18±3.50	4.51±0.98
*Robinia pseudoacacia*	Leguminosae	Deciduous	Tree	11.17±2.25	Crystal	Crystal	−	−	5	5	130.1±7.1	131.9±3.7	101.5±5.0	107.5±3.5	9.49±1.41	5.61±3.80
*Rosa xanthina*	Rosaceae	Deciduous	Shrub	0.22±0.02	Crystal	Crystal	−	−	5	5	129.2±5.8	136.1±4.5	82.3±15.1	93.4±9.3	13.52±2.63	16.03±1.56
*Salix psammophila*	Salicaceae	Deciduous	Shrub	1.71±0.26	Film	Crystal	−	−	3	3	107.1±7.7	122.8±3.2	75.6±2.6	96.3±13.2	16.43±2.11	20.72±3.59
*Sophora davidii*	Leguminosae	Deciduous	Shrub	0.42±0.01	Crystal	Crystal	−	+	4	4	127.8±8.7	139.0±4.4	81.8±7.1	106.2±9.6	18.55±3.86	10.82±2.13
*Sophora flavescens*	Leguminosae	Deciduous	Shrub	4.08±0.54	Crystal	Crystal	−	+	4	5	124.6±5.0	138.5±4.2	87.2±9.9	101.5±8.1	27.35±5.20	9.43±5.23
*Spiraea salicifolia*	Rosaceae	Deciduous	Shrub	4.51±1.16	Crystal	Crystal	−	+	3	5	90.6±24.1	142.5±5.4	60.1±13.6	100.0±9.9	59.16±8.35	10.10±0.75
*Stipa capillata*	Gramineae	Deciduous	Herb	2.44±0.22	Crystal	Crystal	−	−	5	5	144.0±2.5	75.6±12.3	110.0±5.3	65.6±5.1	5.67±1.84	
*Tribulus terrester*	Zygophyllaceae	Deciduous	Herb	0.40±0.02	Film	Film	+	+	2	5	102.6±4.2	135.7±5.7	90.1±8.6	89.6±6.1	18.02±2.94	15.48±2.68
*Ulmus bergmanniana*	Ulmaceae	Deciduous	Tree	13.44±2.90	Film	Film	−	−	3	2	50.6±11.2	50.4±9.3	50.3±8.6	45.7±7.8	52.12±2.43	74.53±7.41
*Ulmus pumila*	Ulmaceae	Deciduous	Tree	8.15±1.45	Film	Film	−	−	3	3	63.7±8.6	61.3±9.8	63.2±3.6	64.7±6.5	35.36±1.35	48.85±7.53
*Viburnum dilatatum*	Caprifoliaceae	Deciduous	Shrub	12.37±2.35	Film	Film	+	+	2	3	92.2±15.0	90.7±29.0	61.6±5.5	66.8±7.8	66.13±4.70	36.55±4.15
*Vitex negundo*	Verbenaceae	Deciduous	Shrub	12.86±2.52	Crystal	−	+	+	5	5	72.5±9.6	123.7±3.8	58.1±2.1	82.8±3.8	47.14±4.52	17.92±4.05
*Ziziphus jujuba*	Rhamnaceae	Deciduous	Shrub	3.15±0.63	Film	Crystal	−	−	1	4	77.0±18.0	103.5±7.9	59.5±2.9	63.0±2.0	45.24±13.83	42.18±1.25

The data represent means ± SD. AD and AB indicate adaxial and abaxial surfaces, respectively; “−” in the wax column indicates that epicuticular wax could not be observed because of dense trichomes; “−” in the trichomes column indicates absence, “+” indicates presence.

Leaf sampling was carried out in June and July, 2009. For each species, approximately 200−500 fully-developed and undamaged leaves were collected in the field with a telescoping tree pruning pole (a height of 4.5 m) or a pruning scissors. Approximately equal samples from different directions and heights of the canopy were collected and pooled together in the field to provide a representative sample. Leaf samples were transported to the laboratory in a cool box as soon after cutting as possible, and then leaves were kept in a refrigerator.

### Determination of leaf water drop adhesion

Adhesion was determined as the difference in weight between leaves before and after artificial wetting, expressed on a hemi-surface area basis [1]. Measurements were made separately on the adaxial and abaxial surfaces. Three batches were prepared initially for both each species and each surface. To this end, 15–20 (large) and 30–80 (small) leaves were selected and their fresh weights without added water were determined using an electronic balance (0.0001 g accuracy, FA2004, Shanghai Precision Instruments, Shanghai, China). Next, each leaf was held individually with tweezers and then wetted by submersing it in water for 10 s. After allowing all surplus water to drip-off (usually less than 10 s), each leaf was held horizontally with the adaxial surface up. And then, water on the abaxial surface was carefully blotted off with filter paper prior to measurement, and the leaf was re-weighed. The entire procedure typically took less than 1 min, minimizing water loss by transpiration (prior to wetting) and evaporation (after wetting). Immediately after re-weighing, hemi-surface leaf areas were determined. For broad-leaved species, leaf areas were determined with Image J software (Version 1.46, National Institutes of Health, Bethesda, MD, USA) after scanning (HP Scanjet G2410, HP, Japan). For *Pinus tabulaeformis*, leaf areas were determined according to Eq. (1) [27].
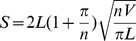
(1)where *L* (m), *n*, and *V* (m^3^) are the average leaf length, number, and volume of *P. tabulaeformis* needles, respectively.

The adhesion of water to the adaxial surface was calculated using Eq. (2),
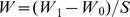
(2)where *W* is the adhesion (g/m^2^), *W_1_* and *W_0_* are the wetted (with the deposition of water) and non-wetted (fresh) weight of the leaf (g), and *S* is the hemi-surface area of the leaf (m^2^).

When measurements were made on the abaxial surface, each leaf was reweighted after carefully removing water on the adaxial surface. However, for *P. tabulaeformis*, *Hippophae rhamnoides*, and *Stipa capillata*, water is difficult to remove from only one side, so the water drop adhesion was the sum of adhesion on both of the adaxial and abaxial surfaces.

### Observation of leaf microstructure and estimation of leaf roughness

The microstructure of the investigated leaves was observed using a scanning electron microscope (JSM–6510LV, JEOL, Tokyo, Japan). For each species, approximately 2×0.5 cm^2^ (broad-leaved species) or 2×0.5/1.0 cm (*P. tabulaeformis* and broad-leaved species with very small leaf) were cut with a razor blade from the middle part of the leaf discarding the mid vein. Next, these samples were mounted on aluminum stubs with double-sided adhesive tape, with adaxial and abaxial surfaces exposed side by side on the same stub. Then the specimens were examined and photographed under at an accelerating voltage of 5–10 kV, a working distance of 10–13 mm, and a magnification of 100–2000.

Leaf roughness on the adaxial and abaxial surfaces was classified into five classes representing the distribution of trichomes, wax structure, the shape of epidermal cells with the method introduced by Sæbø et al. [28] and modified by the authors; this required the use of scanning electron microscope images with a magnification of 100 or 1000 for pubescent and glabrous leaves, respectively. Class 1 has smooth epidermal cells, no trichomes and wax crystals. Class 2 has slightly convex epidermal cells, no trichomes and wax crystals. Class 3 has a coverage of <30% of wax crystals, trichomes or both, convex epidermal cells, and leaves with ridges and hollows. Class 4 has a coverage of 30%−60% of wax crystals, trichomes or both, convex epidermal cells, and a number of ridges and hollows. Class 5 has a coverage of >60% of wax crystals, trichomes or both, very convex epidermal cells, and many ridges and hollows.

### Measurements of contact angles

The advancing contact angles (*θ*) of two liquids, distilled water and diiodomethane (purity ≥99%, Beijing Chemical Reagent Factory, Beijing, China) were measured at room temperature using a JC2000C1 contact angle meter (Zhongchen Science and Technology, Shanghai, China).

Contact angles were determined on adaxial and abaxial surfaces (15 repetitions) by placing the baseline tangent to the area of touch between the solid and the liquid enabled by measuring device software (Version 1.0.0.1, Zhongchen Science and Technology, Shanghai, China) as the average of the contact angles on the right and left sides of the drop. Leaf samples of about 2×0.5 cm^2^ (broad-leaved species) or 2×0.5/1.0 cm (*P. tabulaeformis* and broad-leaved species with very small leaf) were cut with a razor blade from the middle part of the leaf discarding the mid vein; these were mounted on a microscope slide with double-sided adhesive. Droplet volumes of 6 µl or 2 µl of distilled water and 2 µl of diiodomethane were selected based on the unit leaf area, the properties of the liquids, and the effect of droplet volume on the contact angle (i.e., contact angles were independent of the droplet volume for volumes between 1 and 10 µl [13].

### Theoretical background and calculations based on contact angle determination

Measurement of the contact angle with a set of liquids with different surface free energies and dispersive and polar components on a given solid surface is generally considered the most practical way to obtain a measure of the solid surface free energy [29]. The solid-liquid interfacial tension, at the triple line between the solid, liquid and vapor, can be expressed through Young's equation (3) in the absence of spreading pressure [25]:
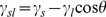
(3)where *γ_s_* and *γ_l_* are the surface free energy of the solid and liquid (mJ/m^2^), respectively, and *θ* is the contact angle (°).

A great contribution for calculation of solid surface free energy has appeared with Fowkes' treatment that suggested that surface free energy could be divided into two components [23]:
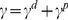
(4)where *γ* ^d^ and *γ* ^p^ are the dispersive and polar components (mJ/m^2^), respectively.

According to Owens-Wendt [24], the interfacial tension between a liquid and a solid can be evaluated by the geometric mean equation (5):
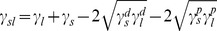
(5)where *γ_s_* ^d^ and *γ_l_* ^d^ are the dispersive components of surface free energy of the solid and liquid (mJ/m^2^), respectively, and *γ_s_* ^p^ and *γ_l_* ^p^ are the polar components of surface free energy of the solid and liquid (mJ/m^2^), respectively.

Combining Eqs. (5) and (3) yields:
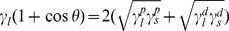
(6)


Finally, the work-of-adhesion for water (*W_a_*) was determined following the Young-Dupré equation (7) [26]:
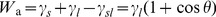
(7)


For all the surfaces evaluated, the surface free energy and its components, i.e. the dispersive and polar components, as well as the work-of adhesion for water were calculated, considering the *γ*, *γ* ^p^ and *γ* ^d^ are 72.8, 51.0, and 21.8 mJ/m^2^ (distilled water) and 50.8, 2.3, and 48.5 mJ/m^2^ (diiodomethane) [12], [29].

### Data analysis

Analysis of variance (ANOVA) and *t* test was undertaken using SPSS (Version 19, IBM, Chicago, IL, USA) statistical packages to estimate the differences in adhesion and contact angle of water among the 60 species, between the pubescent and glabrous leaves, between leaves with wax crystals and wax films, and between the adaxial and abaxial surfaces for each species. When ANOVA indicated significant differences among species, the pairs of species that exhibited significant differences were determined using Tukey's multiple means comparison tests. The relationships between variables were assessed with regression procedures. A given effect was assumed significant at *p*<0.05.

## Results

### Leaf water drop adhesion

Adhesion of water droplets to a leaf differed considerably among species (ANOVA, *p*<0.001) and between adaxial and abaxial surfaces of the same or a different species' leaf (Table 1). Adhesion on the adaxial and abaxial surfaces ranged widely from 4.09 (*Astragalus adsurgens*) to 88.87 g/m^2^ (*Carduus nutans*), with an average of 33.41 g/m^2^, and from 0.72 (*C. nutans*) to 93.35 g/m^2^ (*Agrimonia pilosa*), with an average of 25.01 g/m^2^, respectively. The combined values for adaxial and abaxial surfaces in a single species ranged from 5.67 (*S. capillata*) to 159.59 g/m^2^ (*A. pilosa*). Among the 60 species analyzed (excluding *Artemisia desertorum*, *P. tabulaeformis*, and *S. capillata*), the adhesion was significantly greater on the adaxial surface than on the abaxial surface for 24 species (Table 1, *t*-test, *p*<0.05). Nine species had greater adhesion on the abaxial surface than on the adaxial surface (Table 1, *t*-test, *p*<0.05). No significant difference in adhesion was found between the adaxial and abaxial surfaces for 24 species (Table 1, *t*-test, *p*>0.05).

Leaves with wax films had considerably higher adhesion (45.34±23.22 g/m^2^) than those measured for leaves with wax crystals (18.15±15.70 g/m^2^). However, the difference in adhesion between pubescent (26.18±24.24 g/m^2^) and glabrous (32.31±21.39 g/m) leaves was not significant. Additionally, the leaves with wax crystals, e.g., *Ixeris polycephala* and *Quercus wutaishanica*, and trichomes, e.g., *A. adsurgens* and *Lonicera hispida*, had different levels of adhesion. Finally, closely-related species showed significant differences in levels of adhesion, such as these three groups: *Artemisia gmelinii*, *Artemisia dubia*, and *A. desertorum*; *Cynanchum chinense* and *Cynanchum komarovii*; *Populus davidiana* and *Populus simonii*.

### Leaf contact angle, surface free energy and work-of-adhesion for water

Both for adaxial and abaxial surfaces, water exhibited higher values for the contact angle than those of diiodomethane (Table 1). Of the 60 species analyzed, the contact angle differed significantly among species (ANOVA, *p*<0.001) and between adaxial and abaxial surfaces (Table 1). Leaf contact angle on the adaxial and abaxial surfaces ranged widely from 50.6° (*Ulmus bergmanniana*) to 144.0° (*S. capillata*), 40.8° (*Hyoscyamus pusillus*) to 110.0° (*S. capillata*) and from 44.6° (*Ostryopsis davidiana*) to 142.5° (*Spiraea salicifolia*), 39.8° (*P. tabulaeformis*) to 116.7° (*A. dubia*), of water and diiodomethane, respectively. The presence of wax crystals or trichomes led to higher contact angles as compared with leaves with wax films or glabrous leaves (Table 1).

**Figure 1:**
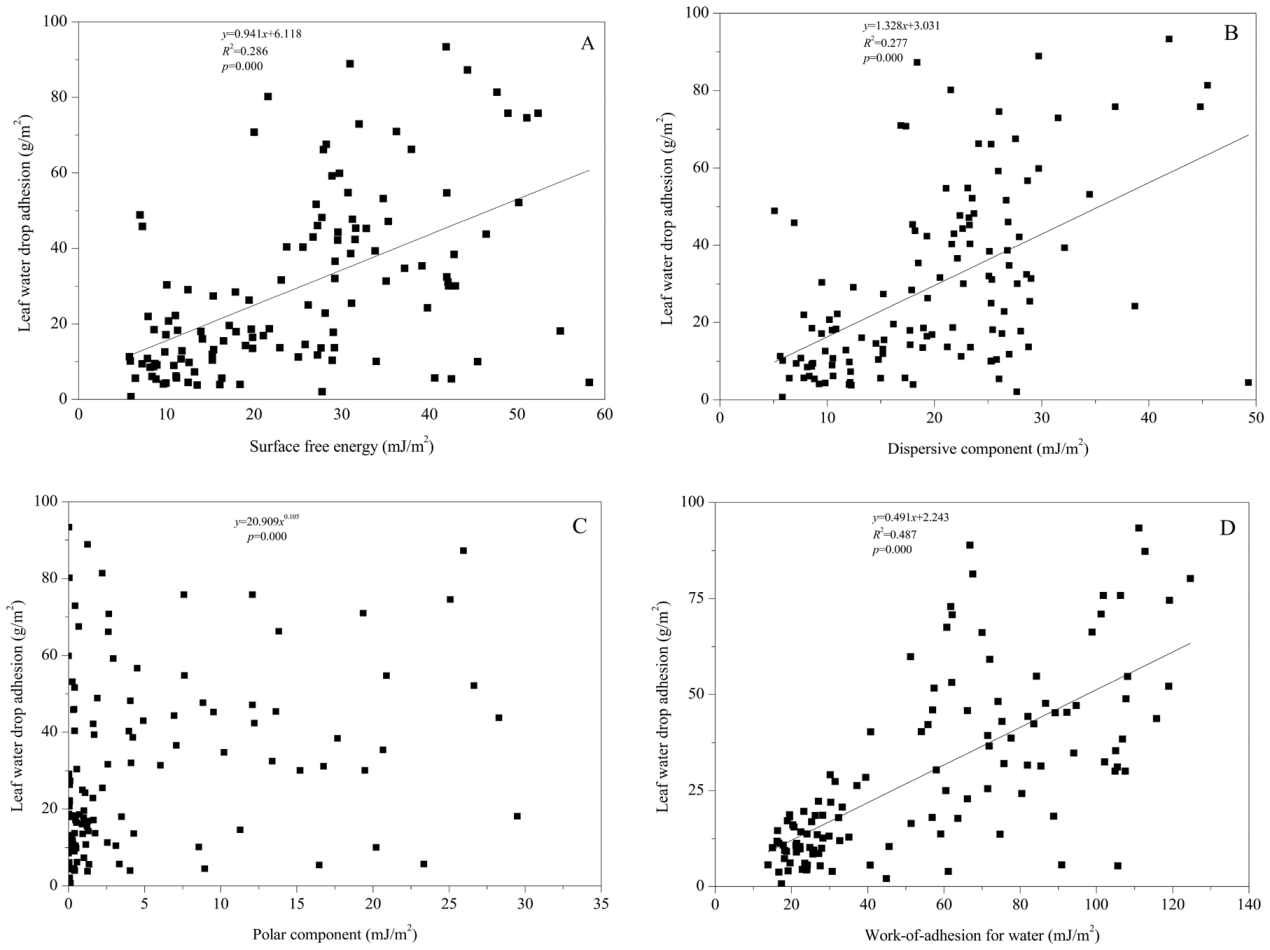
Relationships between leaf water drop adhesion and surface free energy (A), dispersive component (B), polar component (C) and work-of-adhesion for water (D).

For the analyzed species, the surface free energy differed among the species and between abaxial and adaxial surfaces, by 10- and 5-fold, respectively, chiefly as a result of the corresponding Lifshitz van der Waals component, i.e., the dispersive component (Fig. 1). Leaves with wax films had a slightly higher surface free energy and an increased work-of-adhesion for water as compared with the leaves with wax crystals. Similarly, pubescent leaves had higher surface free energy and work-of-adhesion for water than glabrous leaves. Regardless of the wax structure and presence or absence of trichomes, leaves had a higher leaf surface free energy and work-of-adhesion for water on the adaxial leaf sides than those of abaxial surfaces.

### Contacting states of water on some typical plant leaf surfaces

**Figure 2:**
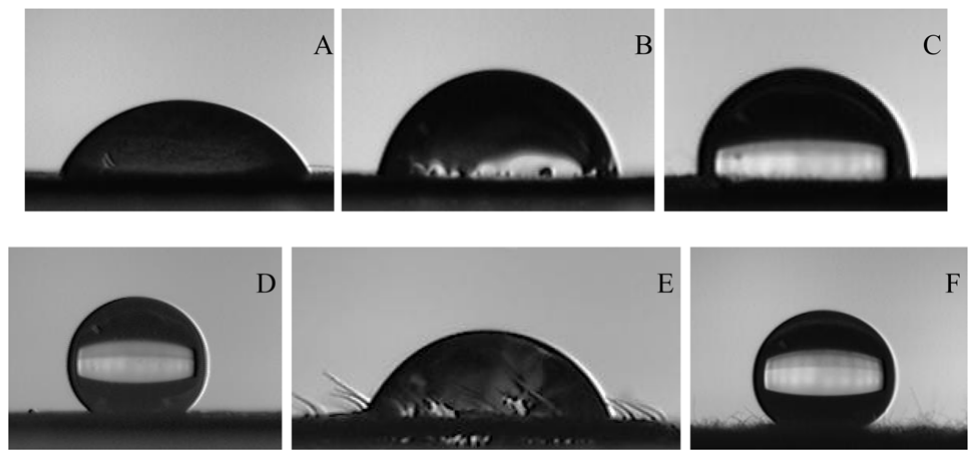
Side views of 6 µl droplets of distilled water placed on some typical plant leaf surfaces. A: abaxial surface of *Agrimonia pilosa*, *θ* = 58.2±8.2°; B: adaxial surface of *Populus simonii*, *θ* = 80.0±11.9°; C: adaxial surface of *Carduus nutans*, *θ* = 94.7±9.2°; D: abaxial surface of *Sophora davidii*, *θ* = 139.0±4.4°; E: the adaxial surface of *Lonicera hispida*, *θ* = 67.0±20.9°; F: the abaxial surface of *Anemone vitifolia*, *θ* = 141.0±4.1°).

When analyzing the patterns of water drop adhesion on leaves, we noticed that the investigated plants appeared to have a broad spectrum of water retained on leaf surfaces. These varied from a continuous layer of water films (e.g., the adaxial and abaxial surfaces of *U. bergmanniana*, *Ulmus pumila*), to large or small patches of water (e.g., the adaxial and abaxial surfaces of *A. pilosa*, the adaxial surface of *C. nutans*, the adaxial and abaxial surfaces of *L. hispida*), to small spherical droplets (e.g., the adaxial and abaxial surfaces of *Poa sphondylodes* and *Sophora davidii*). To further understand the liquid-solid contacting, six leaves with different contact angles were selected (Fig. 2). The contact angles for the abaxial surface of *A. pilosa*, and adaxial surface of *P. simonii* and *L. hispida* leaves were less than 90° and the spreading droplets covered a comparatively large area of the leaf surface (Fig. 2A, 2B, and 2E). Contact angles for the abaxial surface of *S. davidii* and *Anemone vitifolia* exceeded 130° and only a small area of the surface was covered by rounded droplets (Fig. 2D and 2F). The contact angle for the adaxial surface of *C. nutans* was of intermediate magnitude; droplet shape and coverage were also intermediate (Fig. 2C).

### Leaf water drop adhesion and leaf roughness

**Figure 3:**
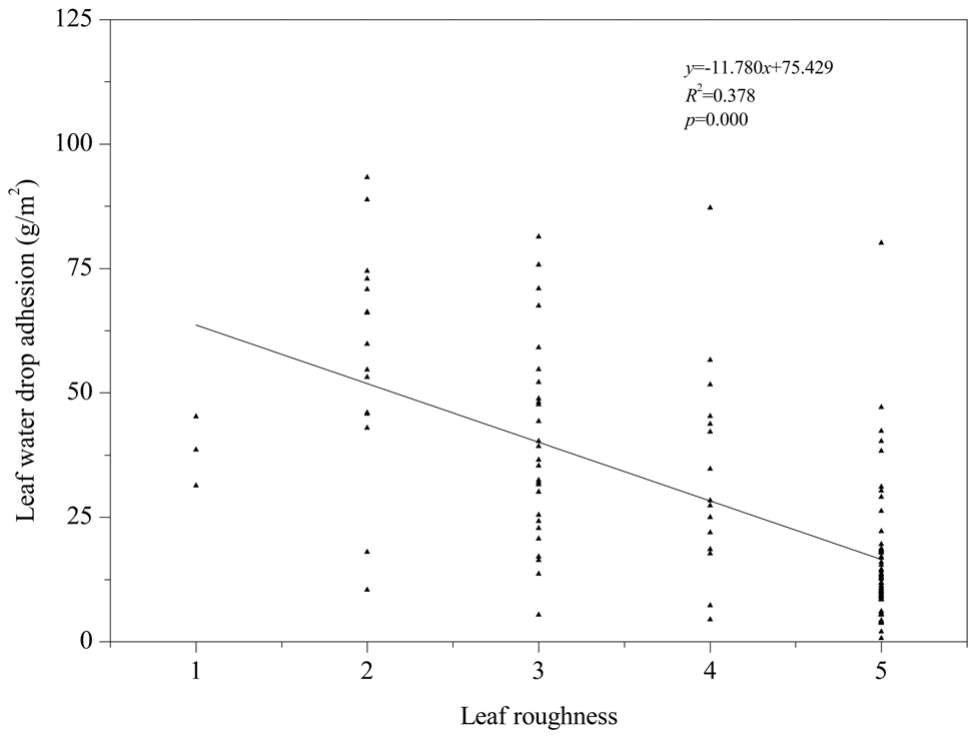
Relationship between leaf water drop adhesion and leaf roughness.

A strong negative correlation (*r* =  −0.615, *p* = 0.000, Fig. 3) was observed between adhesion and leaf roughness. However, there were some obvious outliers in this data series, i.e., (i) the leaf surface had lower roughness and lower adhesion, e.g., the adaxial surface of *Erodium stephanianum* and *Tribulus terrester*, both surfaces of *Salix psammophila* and *P. simonii*; (ii) the leaf surface had high roughness and high adhesion, e.g. the abaxial surface of *Betula dahurica*, *H. pusillus*, *O. davidiana* and the adaxial surface of *C. komarovii* and *Vitex negundo*.

### Leaf water drop adhesion and surface free energy, its components, and work-of-adhesion for water

Adhesion was positively correlated with surface free energy (*r* = 0.535, *p* = 0.000, Fig. 1A), its dispersive component (*r* = 0.526, *p* = 0.000, Fig. 1B), and work-of-adhesion for water (*r* = 0.698, *p* = 0.000, Fig. 1D). However, a significant power correlation was found between adhesion and the polar component of surface free energy (*p* = 0.000, Fig. 1C).

## Discussion

### Comparisons of leaf water drop adhesion

Previous studies have shown that adhesion varies among species. Adhesion of 18 species of clover ranged between 130 and 360 g/m^2^ [8]. Tanakamaru et al. [30] reported on the retention of water drops in young and old leaves of barley (*Hordeum vulgare*), and they obtained a value of 56 and 128 g/m^2^, respectively. Haines et al. [17] found that the adhesion of six investigated species ranged from 39 to 295 g/m^2^. Wohlfahrt et al. [1] compared nine dominate species in Stubai Valley and determined that retention of water drops by leaves ranged from 44.9 to 414.8 and from 13.2 to 314.0 g/m^2^, using the spraying and submersion methods, respectively. The measurements of adhesion in this study of 4.09–88.87 g/m^2^ (adaxial surface), 0.72–93.35 g/m^2^ (abaxial surface), and 5.67–159.59 g/m^2^ (both sides) are much lower than those obtained by Bradley et al. [8], Wohlfahrt et al. [1], and Haines et al. [17]. The methods used may have led to this discrepancy, because some researchers used the spraying method while others used submersion. The results of Wohlfahrt et al. [1] indicated that spraying was much more effective in wetting phytoelement surfaces. Calder et al. [31] found that the wetting rates were higher for drops of small size, but *in situ* observations did not provide clear evidence related to which method is preferred for determining adhesion rates [1].

**Figure 4:**
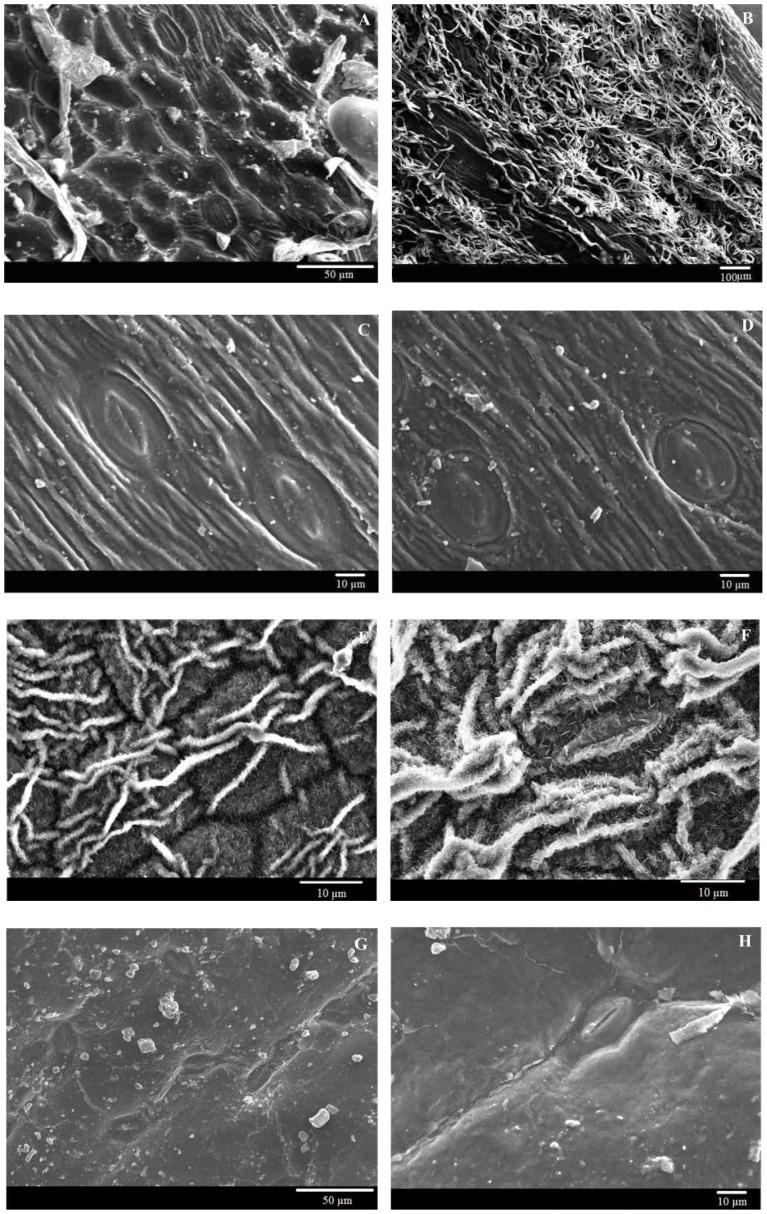
Scanning electron microscope images of the adaxial (A, C, E, G) and abaxial (B, D, F, H) surfaces of *Artemisia gmelinii*, *Artemisia desertorum*, *Cynanchum chinense*, and *Cynanchum komarovii*, respectively.

Closely-related species showed different adhesion rates such as the three groups of *A. gmelinii*, *A. dubia*, and *A. desertorum*; *C. chinense* and *C. komarovii*; *P. davidiana* and *P. simonii*, possibly because of differences in leaf structure between species in the same genus. The presence of trichomes or wax crystals may explain the lower retention of water drops on leaves of *Trifolium pretense* compared with *Trifolium repens* [1], and the smaller leaf water retention in young leaves when compared with old leaves [30]. This can be ascribed to two main factors, i.e. the roughness provided by a dense layer of trichomes or crystals [12], [15], [32], [33], and hydrophobic properties of wax components [12], [34]. The abaxial surface of *A. gmelinii* (Fig. 4B), and the adaxial and abaxial surfaces of *C. chinense* (Fig. 4E and 4F) were densely covered with trichomes or wax crystals, respectively, resulting in lower rates of adhesion. However, both the wrinkled structure on the adaxial surface of *A. gmelinii* (Fig. 4A) and both sides of *A. desertorum* (Fig. 4C and 4D), and the smooth surfaces of *C. komarovii* (Fig. 4G and 4H), could lead to a high rates of adhesion. The following section discusses in detail the effects of leaf roughness induced by trichomes or wax crystals on adhesion.

### Effects of surface roughness on leaf water drop adhesion

An increase in surface roughness sometimes inhibits the spread of droplets on leaf surfaces. A well-known example of this is the lotus leaf, which has microscale roughness created by many 5–10 µm papillae and nanoscale roughness created by three-dimensional epicuticular waxes that create a very rough superhydrophobic surface [15], [35]. Puente and Baur [19], Massinon and Lebeau [20] and Wohlfahrt et al. [1] observed that plants with epicuticular wax crystals or trichomes retained fewer droplets, and they postulated that this was the result of microroughness. Plants are commonly categorized as easy-to-wet or difficult-to-wet based on the absence or presence of epicuticular wax crystals or trichomes [4], [5], [12], [22]. Hence, a strong correlation would be expected between the observed surface roughness and the measured adhesion. Our observations confirmed this hypothesis. The fact that a strong correlation exists between these two parameters demonstrated that surface roughness contributed significantly to adhesion. The leaves of *A. gmelinii* (the adaxial surface with a dense layer of trichomes), and *A. desertorum* (glabrous), and of *C. chinense* (wax crystals) and *C. komarovii* (wax films) can be taken as examples. Both leaves without trichomes or wax crystals had higher retention rates for water drops; however, the leaves with trichomes or wax crystals had a much lower rate of adhesion. Studies have shown that the hierarchical level of structuring may occur in association with the general shape of the epidermal cells, cuticular folds, the presence of trichomes and epicuticular wax crystals [36]. The nano-structure of papillae, trichomes and wax crystals can also have a major effect on surface roughness [22].

**Figure 5:**
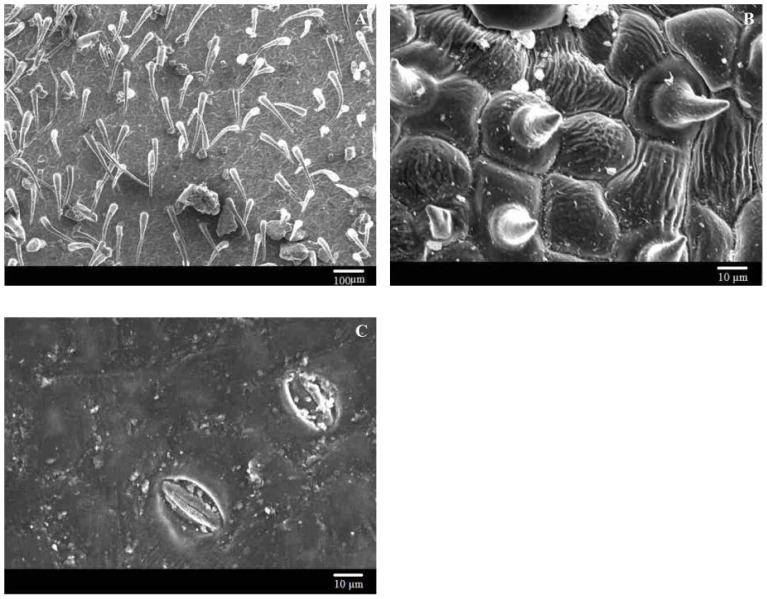
Scanning electron microscope images of the abaxial surface of *Hyoscyamus pusillus* (A), the adaxial surface of *Vitex negundo* (B) and *Populus simonii* (C).

During this study, we also observed some obvious outliers, such as *H. pusillus, V. negundo*, and *P. simonii*. The contact angle measurements suggested the leaf surfaces were easy-to-wet, but they had significant difference in adhesion. Holloway [18] postulated that the enhanced wetting observed in some plant species with “open” trichome patterns caused by capillary action. Brewer et al. [37], [38] found three different types of trichome interactions with water. The effects of trichomes on adhesion depend on trichome density, and/or structure, and the presence or absence of wax, because these factors influence the patterns of interaction between the leaf surface and water droplet [12], [37]–[39]. The abaxial surface of *H. pusillus* is densely covered by trichomes (Fig. 5A), and the adaxial surface of *V. negundo* has trichomes and wrinkled cell surfaces (Fig. 5B) that confer high leaf roughness. We propose that the high adhesion of the abaxial surface of *H. pusillus* and the adaxial surface of *V. negundo* is caused by capillary action or the “segregating strategy” (e.g., Fig. 2E), that segregated water into patches based on water drawn along the trichomes or rugose surface. However, the low roughness and low adhesion of the adaxial surface of *P. simonii* (Fig. 5C) may occur because roughness describes only the physical interaction between the drop and the surface, whereas droplet adhesion is also influenced by chemical attractive and repulsive forces between the two [10], [18], [22].

### Effects of surface free energy, its components and work-of-adhesion for water on leaf water drop adhesion

Adhesion stems from the interaction between the liquid and leaf surfaces, and this interaction is both physical and chemical [9], [10], [18], [22]. Fernández et al. [9] demonstrated that chemical modification of leaf wax in wheat induced by adding phosphorus in soil could drastically change the measured contact angles, and thus the surface free energy, its components, and work-of-adhesion for water. Surface free energy, its components and work-of-adhesion are physico-chemical properties of all materials (solids, liquids, and gases). Different plant leaves have different chemical compositions, affecting the surface free energy, its components, and work-of-adhesion for water. In this study, we observed that a continuous film or patches of water covered the surfaces of many species with higher surface free energy and work-of-adhesion. However, some species with lower surface free energy and work-of-adhesion (e.g., *A. adsurgens*, *Caragana korshinskii*, *I. polycephala*) could not be wetted when immersed in water. This confirmed the effects of leaf surface free energy as well as the work-of-adhesion for water on retention of water by leaves. Whether the water droplet can adhere to leaf surfaces on the droplet properties (e.g., diameter and kinetic energy) and on the impacted surfaces (e.g., dry or wetted) [40].

Our results included the analysis of the different patterns of water drop adhesion on leaves and the significant correlations between adhesion and surface free energy, its components, and work-of-adhesion for water. These results imply that the degree to which and amount of water that can be attached to leaf surfaces was determined by the surface free energy, its components and work-of-adhesion for water, especially the latter. In this regard, the estimation of the work-of-adhesion for water constitutes an easy and valuable tool that can be used to quantify the degree of adhesion for a particular plant surface [9].

## Conclusions and Suggestions

The adhesion of water drops to leaves differed considerably among species, between leaf sides, and between leaves with wax films and wax crystals. In general, more water adhered to the adaxial surface than on the abaxial surface. Likewise, more water adhered to leaves with wax films compared with those with wax crystals. However, the rates of adhesion were not significantly different between pubescent and glabrous leaves.

Our results indicated that the retention of water drops by leaves was influenced by the physical and physico-chemical properties of leaves, including leaf roughness, surface free energy, dispersive component, polar component, and work-of-adhesion for water as predicted by these strong relationships, especially the work-of-adhesion for water. However, there were some outliers, such as the abaxial surface of *H. pusillus* and the adaxial surface of *V. negundo*, both with high roughness and high adhesion. This discrepancy may be the result of capillary action, with water drawn along the trichomes or rugose surface.

The strong correlation between adhesion and work-of-adhesion for water implies that the estimation of the work-of-adhesion for water can provide a useful method to quantify the amount of adhesion to be expected for any species. Meanwhile, because increases in leaf roughness or decreases in surface free energy and work-of-adhesion for water can lower leaf water deposition, changes in vegetation may impact the discharge of water from watersheds because humans have consistently altered vegetation in watersheds by replacing existing species with new species. Hence, the effects of leaf surface properties (leaf roughness, surface free energy, its components, and work-of-adhesion) on hydrological processes provide an important avenue for further study.
